# The relationship between Connexin 43 (Cx43) and partner protein, human discs large homologue-1 (Dlg1) during wound closure in keratinocytes

**DOI:** 10.1007/s00441-025-04030-9

**Published:** 2026-02-18

**Authors:** Harry Scott, Li Dong, Andrew Stevenson, Patricia E. Martin, Sheila V. Graham

**Affiliations:** 1 School of Infection and Immunity. College of Medical, Veterinary and Life Sciences, MRC-University of Glasgow Centre for Virus Research, University of Glasgow, Garscube Estate, Glasgow, G61 1QH Scotland, UK; 2 Department of Biological and Biomedical Sciences, School of Health and Life Sciences, Glasgow Caledonian University, Cowcaddens Road, Glasgow, G4 0BA Scotland, UK; 3 Bone-Muscle Research Center, The University of Texas at Arlington, Arlington, TX USA

**Keywords:** Gap junctions, Connexin 43, Discs large homologue-1 (Dlg1), Keratinocytes, Wound closure

## Abstract

Plasma membrane gap junctions, formed by connexin proteins, are responsible for direct intercellular communication between adjacent cells by allowing the exchange of ions, metabolites, secondary messengers and microRNAs. Connexin 43 (Cx43) is a widely expressed gap junction protein. Cx43 mis-expression is associated with various disease states, including chronic non-healing wounds. Loss of Cx43 from the plasma membrane is important to allow wound resolution. Cx43 expression increases, and gap junctions reform, upon wound closure. Cx43 directly interacts with Dlg1, a membrane-associated guanylate kinase (MAGUK) protein, which controls cell shape and polarity. In this study, AlphaFold3 modelling predicted an interaction between amino acids 263–269 and 302–320 of Cx43 and the SH3, HOOK and GUK domains of Dlg1. Since Dlg1 is required for maintaining Cx43 protein levels at the plasma membrane, we hypothesised that Dlg1 may regulate wound healing via its functional interaction with Cx43. Cx43 and Dlg1 relocated to the cytoplasm of cells at the wound edge in scrape wound assays and co-located in pseudopodia of cells migrating towards the wound edge in live cell imaging experiments. Scratch wound assays carried out in HaCaT cells treated with siRNA targeting Dlg1 showed that Dlg1 depletion resulted in reduced wound closure rates. This was not due to changes in cell migration, suggesting that Dlg1 controlled cell proliferation. The data demonstrate that Cx43 and Dlg1 both contribute to wound closure, but that Dlg1 has other crucial functions in migrating cells separate from its role in maintaining Cx43 at the plasma membrane.

**Supplementary Information:**

The online version contains supplementary material available at 10.1007/s00441-025-04030-9.

## Introduction

Gap junctional intercellular communication (GJIC) is critical for maintaining cellular homeostasis (Chanson et al. 2018; Evans 2015). Gap junctions (GJs), formed by connexins (Cxs), permit cell-to-cell transfer of ions and metabolites (< 1 kDa) and control cellular proliferation (Laird 2006). GJIC is tightly regulated through connexin protein post-translational modification, membrane trafficking, internalisation and degradation (Leithe et al. 2018). GJs comprise two juxtaposed hexamers of connexin (Cx) proteins (Aasen et al. 2018). There are 21 Cxs, each with four trans-membrane domains, a short intracellular N-terminus and an intracellular C-terminus (Aasen et al. 2018). Cxs have a brief life cycle (1.5–5 h). They are synthesised and form hemichannels in the trans-Golgi network (Cx43) or, for other Cxs, in the endoplasmic reticulum-Golgi intermediate compartment. Connexons, formed of six connexins, are trafficked to the plasma membrane in a closed state and inserted at the edges of existing multi-GJ “plaques” (Musil and Goodenough 1993). Cxs are recycled from the centre of GJ plaques and are degraded by the endosomal/lysosomal pathway but can also be degraded by the proteasome (Berthoud et al. 2010; Epifantseva and Shaw 2018; Su and Lau 2014; Totland et al. 2020; Falk et al. 2014). Connexin membrane trafficking and function are highly susceptible to changes in the cellular environment (Leithe et al. 2018).

Cx43 is the most widely expressed connexin with key roles in skin physiology. Changes in Cx43 localisation and post-translational modification have been implicated in epithelial wound healing (Lorraine et al. 2015). Cx43 is down-regulated in the early stages of wound closure in normal keratinocytes but is over-expressed at the wound edge of chronic non-healing wounds in non-diabetic and diabetic patients (Lorraine et al. 2015). Cx43 is one of the largest connexins with a long intracellular C-terminus (Ct) which can be post-translationally modified (e.g. phosphorylation) to affect protein–protein interactions (Aasen et al. 2018). Cx43 has been shown to interact with a wide range of proteins (Singh and Lampe 2003) including the multi-domain proteins of the membrane associated-guanylate kinase (MAGUK) family, which have roles in cell signalling cascades and cell morphology organisation (Subbaiah et al. 2011). The most well-documented interaction of Cx43 with a MAGUK protein is with ZO-1. The last four amino acids of the Cx43-Ct domain are necessary for ZO-1 interaction (Giepmans and Moolenar 1998; Toyofuku et al. 1998; Ambrosi et al. 2016; Gourdie et al. 2006). However, ZO-1 binds as a dimer and up to 19 amino acids of the Cx43 C-terminus may be involved (Fanning et al. 2007; Xiao et al. 2011). Binding of ZO-1 to Cx43 limits the rate at which undocked hemichannels in the plasma membrane incorporate into GJ plaques (Palatinus et al. 2012; Rhett et al. 2010, Hunter et al. 2005). Importantly, the Cx43 mimetic peptide, ACT1, promotes phosphorylation of Cx43, inhibits binding with ZO-1 and stimulates GJIC. ACT1 has shown therapeutic promise in enhancing wound closure, with Granexin gel (which contains ACT1) being granted orphan drug designation for cutaneous radiation injury. Clinical trials for other conditions are ongoing (Ghatnekar et al. 2009; Ongstad et al. 2013; Jiang et al. 2019).

Another MAGUK protein, the human homologue of *Drosophila* discs large protein-1 (Dlg1), has been shown to interact with Cx32 (Duffy et al. 2007, Stauch et al. 2012). We have shown that Dlg1 also interacts directly with Cx43 and controls Cx43 location at the membrane of keratinocytes (Macdonald et al. 2012; Sun et al. 2015; Scott et al. 2023). In normal epithelial cells, Dlg1 is found predominantly at intercellular contact sites where it is part of the Scribble complex (Stephens et al. 2018). Dlg1 binds E-cadherin to link to α- and β-catenins and the actin cytoskeleton and, through its protein partners, integrates signalling cascades (Firestein and Rongo 2001; Reuver and Garner 1998; Wu et al. 1998). Dlg1 has roles in cell membrane integrity and adhesion, cell proliferation and differentiation and is a key regulator of cell polarity (Bonilha and Rodriguez-Boulan 2001; Golub et al. 2017; Knoblich 2008; Müller et al. 1995; O’neill et al. 2011; Woods et al. 1996). Recent data also implicate Cx43 in control of cell polarity, suggesting a possible synergy between Dlg1 and Cx43 (Pun et al. 2024). There is some evidence of a role for Dlg1 in promoting cell migration during wound healing. For example, siRNA depletion of Dlg1 in H1703 murine astrocytes resulted in a 20% reduction in cell migration into the wound area compared to control siRNA treatment (O’neill et al. 2011). Dlg1’s interaction with protein kinase C-alpha could promote cell invasion in cancer cell lines (O’neill et al. 2011), raising questions as to whether it could stimulate cell migration in normal keratinocytes.

Cx43 binds the N- and C-termini of Dlg1 through its C-terminal domain. Importantly, the final four amino acids of Cx43-Ct that are required for ZO-1 binding do not mediate the Dlg1 interaction (Macdonald et al. 2012). This was confirmed here by AlphaFold3 modelling, which indicated Cx43 amino acids 263–269 and 302–320 as the two major interacting sites in the Dlg1 C-terminus. Aside from physical interaction, Dlg1 has a positive functional interaction with Cx43 because Dlg1 knockdown in HaCaT keratinocytes leads to Cx43 relocation to the cytoplasm, a reduction in Cx43 levels and in GJIC (Scott et al. 2023). Our data indicate that Dlg1 is required to maintain the location of Cx43 on the plasma membrane and to protect it from lysosomal degradation. In this study, using in vitro wound closure experiments, we examined the hypothesis that Dlg1 might influence Cx43 intracellular fate during wound healing. A proportion of both proteins moved from the plasma membrane to co-localize in the cytoplasm, but their movement was not synchronised. Dlg1 followed Cx43 in relocalising from the plasma membrane and back to it during the wound closure process. Keratinocyte scratch wound assays showed that Dlg1 was required for efficient wound closure rates. This was not due to changes in cell migration, suggesting that Dlg1 controlled cell proliferation. Taken together, the data reveal new information regarding the relationship between Cx43 and Dlg1 during wound closure.

## Materials and methods

### AlphaFold3 modelling

AlphaFold3 models were generated using the AlphaFold3 online server (https://alphafoldserver.com/welcome) (Jumper et al. 2021). Sequences for the human Cx43 C-terminus (amino acids 232–382) and human Dlg1 C-terminus (amino acids 569–764) were used together as input. Five models of the interaction between Cx43 and Dlg1 were generated, with a maximum of three recycles. The top-ranked model of Cx43 and Dlg1 interaction was evaluated using a pLDDT plot (a per-residue confidence score, from 0 = low confidence to 100 = high confidence) and PAE values (confidence in the placement of domains relative to other domains in a 3D space, from 0 = low error to 30 = high error). Results were exported to Chimera X software for analysis (Pettersen et al. 2021). Contacts were set at a maximum distance of 4 Å between atoms.

### Cell culture

HaCaT (Boukamp et al. 1988) and HEK293 cells were cultured in Dulbecco’s Modified Eagle’s Medium (DMEM) (Thermo Fisher, Scientific UK, catalogue # 31,966–021) supplemented with 10% (v/v) FBS (Thermo Fisher Scientific, UK, catalogue # 10,270–106) and 250 units/ml penicillin–streptomycin (Thermo Fisher Scientific, UK, catalogue # 15,140–122). Cells were maintained in a humidified incubator with 5% CO_2_ at 37 °C. Cells were detached from plates using 0.05% trypsin–EDTA (Thermo Fisher Scientific, UK, catalogue # 25,300–054) and passaged three times a week at either a 1:5 or a 1:10 dilution depending on growth rate.

### Plasmids

To generate a plasmid containing the sequence of human Cx43 with a C-terminal mCherry tag, we designed primers to clone human Cx43 into the pmCherry-N1 vector (Clontech) (primer sequences shown in Table 1). Primers were synthesised by Eurofins, UK, and used to amplify Cx43 from pCR3.1Cx43 using a High Fidelity PCR mix (Roche, UK Catalogue # 03310256103). The PCR product was purified using a PCR cleanup kit (Qiagen, Germany) and cloned into the pmCherry-N1 vector as described in the Clontech In-Fusion kit manual (Thermo Fisher Scientific, UK, catalogue # NC1454739). Resulting clones were sequenced by Eurofins to confirm the presence of the Cx43 gene.

**Table 1:** Primers used for cloning of Cx43 into the pmCherry-N1 vector

Primer	Sequence
Cx43 forward	CTCAAGCTTCGAATTCATGGGTGACTGGAGCGCC
Cx43 reverse	GGCGACCGGTGGATCCTCGATCTCCAGGTCATCAGG

The pegfp-C1 mammalian expression plasmid containing the sequence coding for rat Dlg1 with a GFP tag at the N-terminus was kindly gifted by Dr Lawrence Banks and Dr Miranda Thomas at the International Centre for Genetic Engineering and Biotechnology, Trieste, Italy.

### siRNA depletion of Dlg1

For siRNA transfections, cells were cultured as described above in either 6- or 12-well plates until they reached 30–50% confluency, at which point transfection with siRNA against Dlg1 (ON-TARGETplus SMARTpool siRNA, Dharmacon, catalogue # L-009415–00–0010) or control oligonucleotide (siGLO, Dharmacon, catalogue # D-001630–01-0) was carried out using Lipofectamine RNAiMax transfection reagent (Thermo Fisher Scientific, catalogue # 13,778,075) for 24 h according to the manufacturer’s protocol. siRNA sequences targeting Dlg1 are shown in Table 2. The final concentration of siRNA in each well was 40 nM. The siGLO control oligonucleotide was used at 40 nM as a non-target oligonucleotide and to assess transfection efficiency.

**Table 2:** ON-TargetplusSMARTpool siDlg1 target sequences

siDlg1 SMARTpool target sequences siRNA no	Target sequence
J-0009415–05	GAUCGUAUUAUAUCGGUAA
J-0009415–06	CCAUAGAACGGGUUAUUAA
J-0009415–07	GUACUGGUCAACACAGAUA
J-0009415–08	AAAACGAGAUUAUGAGGUA

### Mitomycin C treatment

For determination of optimum mitomycin C (MMC) concentration for HaCaT scratch wound assays, HaCaT cells were treated with MMC (Sigma, UK, catalogue # M4287) and incubated for 5 min. Final concentrations of MMC ranged from 4 × 10^2^ to 4 × 10^−5^ mg/ml. Cells were washed twice in PBS and incubated in fresh medium for 24 h at 37 °C; thereafter, an MMC concentration of 4 × 10^−1^ was used.

### MTT assay

MTT powder (Abcam, Cambridge, UK, catalogue # ab146345) was dissolved in PBS to a concentration of 5 mg/ml and filter sterilised. If required (as for MMC concentration optimisation), MMC treatment was carried out as above in a 96-well plate, with final concentrations ranging from 4 × 10^2^ to 4 × 10^−5^ ug/ml. Medium was removed following 24 h incubation and 50 µl MTT substrate, as well as 50 µl fresh medium was added to each well. Fifty microliter MTT and 50 µl medium was also added to wells containing no cells for background signal controls. The plate was incubated at 37 °C for a further 3 h. To dissolve formazan crystals formed by MTT, 150 µl DMSO (Sigma, UK, catalogue # D1435) was then added to each well and the plate was rotated in the dark for 15 min. Absorbance was measured at a wavelength of 595 nm. Background values from wells containing only 50 µl MTT plus 50 µl medium were subtracted from test samples. Values were then normalised against control HaCaT cells.

### Scratch wound assays

For investigation of Cx43/Dlg1 localisation and protein levels during wound healing, HaCaT cell monolayers were scratch-wounded using a sterile 20 µl pipette tip. For confocal microscopy, cells were grown on coverslips in wells of a 12-well culture plate, and a single scratch wound was made in the confluent monolayers. For western blot analysis, cells were grown to confluence in the wells of a 6-well culture plate, and three parallel scratches were made in each confluent monolayer. Cell medium was removed, and cells were washed twice in PBS before adding fresh medium. Cells were lysed for western blotting at time points of 0, 4, 8, 16 and 24 h, or imaged at these time points using a confocal microscope.

For scratch wound experiments using the Incucyte® S3 Live-Cell Analysis System (Sartorius, Göttingen, Germany), 0.4 × 10^6^ HaCaT cells were seeded in each well of a 6-well plate. There were three treatment groups: HaCaT control, 40 nM siDlg1-treated, or treated with a 100 nM analogue of Gap27 (Faniku et al. 2018) (ZPC: now named AnGap27 (Faniku 2018)). Following 24-h incubation, siRNA treatment was carried out as described above. The AnGap27 treatment group consisted of three biological replicates; all other treatment groups consisted of six biological replicates. Three technical repeats were performed for each biological replicate. Cells were then incubated for a further 20 h before being transferred to wells of an Incucyte® Imagelock 96-well Plate at a fixed cell density of 0.08 × 10^6^ cells per well. Cells were incubated for 4 h to allow attachment to the plate, with the AnGap27 group being treated with 100 nM AnGap27 30 min prior to the 4-h mark. In mitomycin C-treated groups, MMC was added to a final concentration of 4 × 10^−2^mg/ml 5 min prior to wounding. Wounds were created using the Incucyte® Woundmaker Tool according to the manufacturer’s instructions. Cell medium was removed, and cells were washed twice using PBS before adding fresh media. The plate was placed in the live-cell analysis system for imaging. . Images were taken in each well at 0 h and every 2 h thereafter, with the final time point being 24 or 48 h post-wounding. Quantification of wound area in all wells at each time point was performed using the Incucyte® Scratch Wound Analysis Software Module. Relative wound density percentage (RWD%) values were exported and the average RWD% was calculated for each treatment group at each time point.

### Quantification of cell confluence following depletion of Dlg1

Assessment of cell confluence after siDlg1 treatment was performed using the Incucyte® S3 Live-Cell Analysis System. 5 × 10^3^ HaCaT cells were seeded per well in an Incucyte® Imagelock 96-well Plate and were incubated for 24 h at 37 °C. siRNA treatments were carried out as described above and the plate was immediately transferred to the live-cell analysis system for imaging. Images were captured at 0 h and every 2 h thereafter, with the final time point at 72 h. Quantification of cell confluence was performed using the Incucyte® Base Analysis Software (Sartorius). Values were collated and exported to calculate the average confluence values for each treatment group at each time point.

### Live cell tracking of Cx43 and Dlg1

HEK293 cells in a 35 mm plate were transfected with 1 µg Cx43-mCherry, 1 µg Dlg1-GFP DNA (Marziali et al. 2017) and 5 µl Lipofectamine 2000 (Thermo Fisher Scientific, UK, catalogue # 11,668,027). Cells were incubated for 24 h at 37 °C followed by scratch wounding using a sterile 20 µl pipette tip. Cell medium was removed, and cells were washed twice in PBS before adding fresh medium. Cells were then moved to the environmental chamber of a Zeiss LSM 880 Axio Observer confocal microscope (Zeiss, Oberkochen, Germany) which had been pre-incubated at 37 °C, 5% CO_2_. Images were captured every 10 min for a period of 66 h, at which point wound closure was almost 100%. Images were captured using a Zeiss Apochromat 63x/1.4 NA oil DIC M27 lens. A GaAsP photomultiplier tube (PMT) detector was used to capture 4434 × 1584 pixel images as tile scans with three tiles in total. Acquisition parameters for Cx43 (mCherry) were 561 nm (laser, 0.3% power), 578–696 nm (detection wavelength), 0.66 µs (dwell time), 500 (gain) and 0 (offset). Acquisition parameters for Dlg1 (GFP) were 488 nm (laser, 0.6% power), 493–587 nm (detection wavelength), 0.68 µs (dwell time), 749 (gain) and 0 (offset). Pinhole size was set to 1AU for imaging in both channels. A line average of 4 was applied to both channels. A gain of 568 was used for DIC images. Z-stacks were composed of nine slices with 339 nm z-step size.

### Protein extract preparation and western blotting

Plates containing scratch-wounded cells were washed twice in ice-cold PBS before being lysed in 2xBOLT protein loading buffer (Invitrogen, UK, catalogue # B0008). Protein extracts were syringe-passaged through a 22-gauge needle ten times then sonicated in a Sonibath (Kerry Ultrasonics, Hitchin, UK) for 30 s, followed by 30 s on ice three times. This was performed three times. The samples were boiled at 100 °C for 5 min before loading onto a 12% NuPAGE gel (Invitrogen, UK, catalogue # NP0322BOX) and electrophoresed at 200 V for 45 min in 1xMES buffer (NuPAGE MES Running Buffer 20x, Thermo Fisher, UK, catalogue #NP0002). Proteins were transferred to a nitrocellulose membrane using an iBlot™2 transfer system (Invitrogen, UK, catalogue # IB22001) as per the manufacturer’s instructions. Membranes were blocked in 5% (w/v) milk powder in PBST (PBS containing 0.1% (v/v) Tween 20) at room temperature with rotation for 1 h. Blots were incubated with primary antibodies diluted in PBST with 5% (w/v) milk powder with rotation for 1 h at RT or overnight at 4 °C. Antibodies used were a polyclonal antibody C-6219 against Cx43,1:5000 (Sigma, UK, catalogue # C6219), Dlg1 monoclonal antibody 2D11 (Santa Cruz Biotechnology, USA, catalogue # sc9961) and GAPDH antibody clone 6C5 (Biodesign, UK, catalogue # H86504M,) both used at 1:1000 dilution. The blots were washed three times in PBST for 5 min. They were then placed in tubes containing secondary antibodies diluted in PBST 5% (w/v) milk powder and incubated with rotation for 1 h. Secondary antibodies were HRP-linked goat anti-mouse-IgG or goat anti-rabbit-IgG (Pierce, Thermo Fisher Scientific, UK, catalogue # 35,568 and SA535521) used at a 1:2000 dilution. Blots were washed three times in PBST for 5 min before being incubated with ECL Western blot substrate (Pierce, Thermo Fisher Scientific, UK). The blots were exposed to X-ray film (Thermo Fisher Scientific, UK) and processed in a Kodak X-Omat processor. Signal intensities for the Cx43 and Dlg1 bands were quantified and normalised against the signal for the GAPDH loading control.

### Confocal immunofluorescence microscopy

Cells were grown on sterile 13 × 13 mm coverslips until 90% confluent and then washed twice with PBS. Cells were scratch wounded with a pipette tip as described above before being fixed with 100% ice-cold methanol for 10 min at 4 °C then permeabilised in acetone for 1 min followed by three 5-min washes in PBS. Blocking was performed by incubation at room temperature for 1 h with PBS containing 10% (v/v) donkey serum (Sigma, UK, catalogue # D9663). Primary antibodies were a polyclonal antibody against Cx43 (kind gift of Dr Edward Leithe, Oslo, Norway) used at 1:500 and Dlg1 monoclonal antibody 2D11 (Santa Cruz Biotechnology, USA, catalogue # sc9961) used at 1:200. Antibodies were diluted using 5% (v/v) donkey serum in PBS and incubated for either 1 h at room temperature or overnight at 4 °C. Coverslips were washed five times with PBS. Alexa Fluor 488 donkey anti-mouse (Thermo Fisher Scientific, UK catalogue # A21206) and Alexa Fluor 594 donkey anti-rabbit secondary antibodies (Thermo Fisher Scientific, UK catalogue # A-11032 were diluted in 5% donkey serum in PBS at 1:500 and added to the coverslips for 1 h, protected from light. Coverslips were washed five times in PBS, followed by one wash in distilled water. Coverslips were mounted on glass slides with ProLong™ Gold Antifade Mountant with DAPI (Thermo Fisher Scientific, UK catalogue #, P36935). Control samples were coverslips incubated with secondary antibodies alone. Samples were examined using a Zeiss LSM 710 confocal microscope (Zeiss, Oberkochen, Germany) and Zen black software (Zeiss) was used for capturing images. Images were captured using a Zeiss Apochromat 63x/1.4 NA oil lens. A PMT detector was used to capture 1912 × 1912 pixel images. Acquisition parameters for Cx43 (Alexa Fluor 594) were 561 nm (laser, 2% power), 556–697 nm (detection wavelength), 0.68 µs (dwell time), 700 (gain) and 0 (offset). Acquisition parameters for Dlg1 (Alexa Fluor 488) were 488 nm (laser, 2% power), 492–551 nm (detection wavelength), 0.68 µs (dwell time), 452 (gain) and 0 (offset). Pinhole size was set to 1.17AU for Cx43 and 1.42AU for Dlg1. A line average of 4 was applied to both channels.

### Colocalisation analysis

Quantification of colocalisation between Cx43 and Dlg1 was carried out in the Fiji version of ImageJ. Background subtraction was performed for all images by measuring the mean pixel intensity in an area with no staining in each channel and subtracting this value from the image. The wound area was outlined in each image using the freehand tool, creating an ROI containing all cells within the image. The use of the coloc2 plugin provided Manders colocalisation coefficients for each image. Five separate images containing an average of 23 cells each were analysed for the 0 h, 8 h and 16 h timepoints.

### Statistical analysis

The results of scratch wound assays were analysed using a two-way ANOVA to test whether treatment had a significant effect on relative wound density, followed by Tukey’s test to assess differences between individual treatment groups. Analysis of western blot data was carried out using a Student’s *T*-test. Analysis of MTT assay results was performed using a Kruskal–Wallis test followed by Dunn’s post hoc test. In all cases, a *p*-value of < 0.05 was considered statistically significant.

## Results

### AlphaFold modelling reveals three potential sites of interaction between the C-termini of Cx43 and Dlg1

Previously, we showed using confocal immunofluorescence microscopy that Cx43 and Dlg1 co-located on the plasma membrane of non-tumour cervical epithelial cells but were in the cytoplasm of tumour cells derived from these (Macdonald et al. 2012). Proximity ligation assays confirmed that Cx43 and Dlg1 interact in cervical tumour tissues in vivo (Sun et al. 2015). Co-location was also found in normal keratinocytes, fibroblasts and adipocytes in tissues in vivo (Scott et al. 2023). Purified, FLAG-tagged Cx43 C-terminus and GST-tagged Dlg1 proteins interacted directly in vitro (Macdonald et al. 2012), while co-immunoprecipitation experiments confirmed the interaction in three different epithelial cell lines (Scott et al. 2023). GST pull down experiments revealed that the Cx43 C-terminus interacted with the N- and C-termini of Dlg1. Importantly, the interaction did not appear to include the terminal four amino acids of Cx43 (Macdonald et al. 2012) that are required for the well-characterised interaction with another PDZ protein, ZO-1 (Giepmans and Moolenar 1998; Toyofuku et al. 1998). Previous studies have also shown that the C-terminal of Cx32 interacts with the C-terminal of Dlg1 (Duffy et al. 2007, Stauch et al. 2012).

Now we used AlphaFold3 to investigate the possible sites of interaction between Cx43 and Dlg1.

**Fig. 1:**
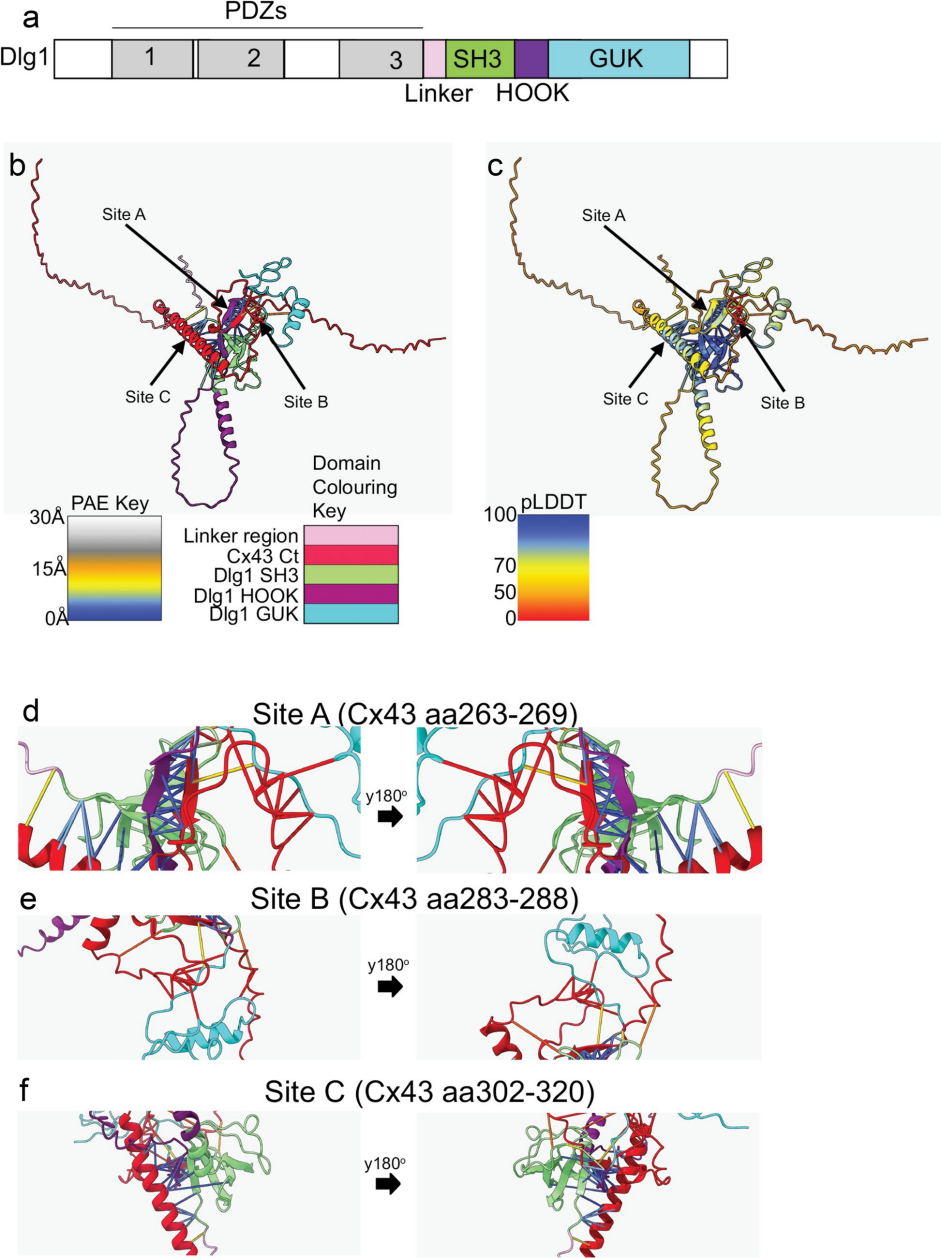
AlphaFold3 modelling of the Cx43 and Dlg1 interaction.** a** Diagram of the domain structure of Dlg1. The horizontal line above the diagram indicates the PDZ domains 1, 2 and 3. **b** AlphaFold3 modelling of sites of interaction between the C-termini of Dlg1 and Cx43. The arrows indicate the three sites of interaction shown in (**d**–**f**). The PAE key that indicates distance of intermolecular contacts is shown beneath the model on the left-hand side. PAE values range from 0 to 30 Angstroms (Å), where low values indicate higher confidence in the relative positions of residues. The Cx43 and Dlg1 domain colour key is shown on the right-hand side. **c** The predicted local distance difference test (pLDDT), a measure of confidence of intermolecular interactions, of the three regions in (**b**) are shown using the colour key beneath the model. High confidence predicted interactions (blue areas) are indicated with arrows. **d** Site A: interaction of Cx43 amino acids 263–269 with the SH3 and HOOK domains of Dlg1. **e** Site B: interaction of Cx43 amino acids 283–288 with the HOOK domain of Dlg1. **f** Site C: interaction of Cx43 amino acids 302–320 with the GUK domain of Dlg1. Rotations around the *y*-axis of 180° are shown for each site (black horizontal block arrows). The PAE key and the domain colour key are the same as those shown in (**b**). Domains of each protein were colored according to the UniprotKB annotation system for each protein (Vasudevan et al. 2011)

A diagram of the domain structure of Dlg1 is shown in Fig. 1a. AlphaFold3 modelling revealed three potential sites of interaction between the C-termini of Dlg1 and Cx43 (Fig. 1b, arrows). Coloured straight lines denote inter-molecular bonds and a colour key for the domains of the two proteins is shown below the figure. Two of the three regions of Cx43 predicted to interact with Dlg1 generally had high predicted local distance difference test (pLDDT) values (sites A and C, values > 70) (Fig. 1c, yellow/blue areas arrowed), while the third region had lower pLDDT values (site B, values < 50) (Fig. 1c, orange areas arrowed). pLDDT values of > 70 indicate high confidence in the predicted structures of the protein. The Dlg1 residues at sites A and C also generally had high pLDDT scores. However, the structure of Dlg1 at site B was predicted with low confidence. Figure 1d–f shows details of the modelling at each putative interaction site indicated in B. As in Fig. 1b, coloured straight lines indicate inter-molecular bonds. Confidence in the correct placement of the interacting residues of the two proteins was quantified by predicted align error (PAE) values ranging from 0 to 30 Angstroms (Å), where low values indicate higher confidence in the relative positions of the two residues. The colour key for PAE values is shown to the left-hand side below Fig. 1b. The density of blue lines in site A (Fig. 1d: amino acids 263–269) indicates a region with many high-confidence sites of contact between the proteins. This region has previously been shown to interact with Drebrin (Butkevich et al. 2004; Singh and Lampe 2003; Ambrosi et al. 2016) and c-Src/PTEN/Csk (Jaraíz-Rodríguez et al. 2017; González-Sánchez et al. 2016). Site C (Fig. 1f: amino acids 302–320) was within an alpha-helical structure that contained high-confidence sites of contact between the proteins. Nuclear magnetic resonance (NMR) spectroscopy experiments have previously shown that Cx43 is capable of forming alpha-helical structures in this region (Sorgen et al. 2004). In contrast, Site B (Fig. 1e: amino acids 283–288) had fewer interactions than Sites A and C and PAE values were higher, suggesting less confidence in the correct placement of the interacting residues. Contact sites were identified between the Cx43 C-terminus and the SH3, HOOK and GUK domains of Dlg1 suggesting that all three domains may be involved in interaction with Cx43. Further in silico validation of the AlphaFold3 model by AlphaFold, ProSA and PROCHECK indicated that interaction sites A and C of Cx43 were in the correct positions relative to the interacting residues of Dlg1 and that the model of the complex was of high stereochemical quality (Supplementary Fig. 1).

### Dlg1 co-locates with Cx43 in keratinocytes during wound closure

**Fig. 2:**
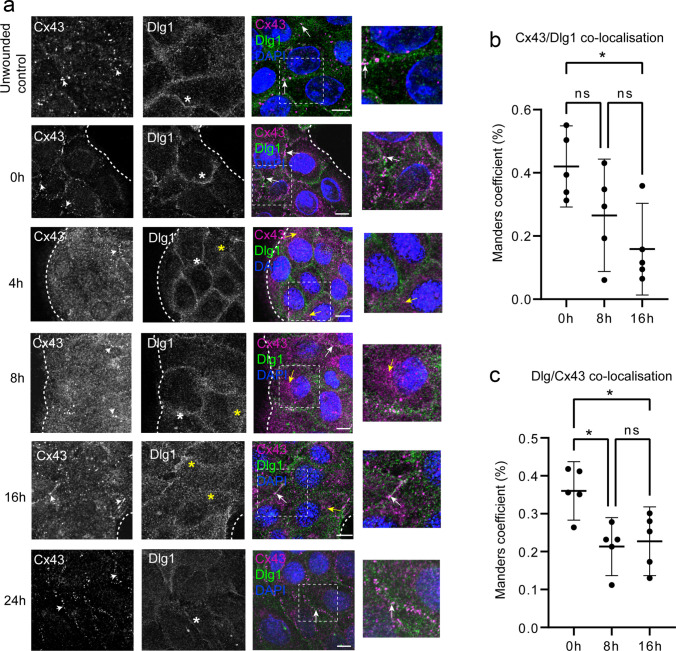
Connexin 43 and Dlg1 are located in the cytoplasm of cells during wound closure. **a** Confocal microscopy images over time showing unwounded control cells and images taken at 0, 4, 8, 16 and 24 h post-wounding of the leading edge of a scrape wound in a confluent HaCaT cell culture. In the merged images, Cx43 is shown in magenta and Dlg1 is shown in green. Blue staining (DAPI) shows the nuclei. Dotted lines indicate the edge of the wounds. White arrowheads indicate examples of gap junction plaques in the Cx43 images. White asterisks indicate examples of Dlg1 at the plasma membrane and yellow asterisks indicate examples of cytoplasmic Dlg1 in the Dlg1 images. In the merged and enlarged merged images, white arrows indicate examples of co-location of Cx43 and Dlg1 on the plasma membrane. Yellow arrows indicate examples of intracellular co-location of Cx43 and Dlg1. Scale bar = 20 µm. **b** Graph of percentage co-localisation of Cx43 with Dlg1 (Manders coefficient) at 0, 8 and 16 h post-scrape wounding. **c** Graph of percentage co-localisation of Dlg1 with Cx43 (Manders coefficient) at 0, 8 and 16 h post-scrape wounding. *p < 0.05, ns, not significant. Calculations were carried out across five images at each time point. Each image had an average of 23 cells

Cx43 and Dlg1 interact in vitro and in vivo in normal keratinocytes (Scott et al. 2023) and in tumour cells derived from cervical epithelia (Macdonald et al. 2012; Sun et al. 2015). Keratinocytes that undergo proliferation and migration to close a wound have similar properties to invasive tumour cells, albeit transiently. Now we investigated the relationship between Dlg1 and Cx43 during epithelial wound closure. HaCaT keratinocytes were grown to confluence on coverslips in wells of a 12-well culture dish and one scrape wound per coverslip was performed on the cell monolayers. Cells were fixed and stained for Cx43 and Dlg1 at set time points post-wounding (Fig. 2a). Confocal immunofluorescence microscopy was used to visualise changes over time in Cx43 (magenta) and Dlg1 (green) location in cells at the leading edge of the scrape wound (Fig. 2a, white dotted lines). In unwounded control cells and at 0 h, Cx43 was found in plaques on the plasma membrane (white arrowheads). Dlg1 was located throughout the cell but was mostly located at the plasma membrane (white asterisks). Both proteins co-located on the plasma membrane in control cells and in cells at 0 h post-wounding (Fig. 2a, white arrows in the merged images). After 4 h, in cells at the wound edge, and just behind the wound edge, Cx43 was mostly lost from the plasma membrane. Diffuse staining was observed in the cytoplasm. Away from the wound edge, cells retained plasma membrane Cx43 (white arrowhead). Dlg1 mostly remained on the plasma membrane (white asterisk), but some Dlg1 was also found in the cytoplasm (yellow asterisk) and some co-located with Cx43 (yellow arrows, merged image). After 8 h, Cx43 was found in the cytoplasm of cells at the leading edge of the wound, but some Cx43 plaques had begun to form at the plasma membrane (white arrowheads). Less Dlg1 was located at the plasma membrane and more could be detected in the cytoplasm (yellow asterisk). There was some co-location of Cx43 with Dlg1 in the cytoplasm (yellow arrow, merged image) and on the plasma membrane (white arrow, merged image). At 16 h, Cx43 was mostly located in plaques on the plasma membrane (white arrowheads), but Dlg1 showed a more cytoplasmic location (yellow asterisks). Co-location of Cx43 and Dlg1 was found on the plasma membrane (white arrow, merged image) and in the cytoplasm (yellow arrow, merged image). At 24 h, the wound had closed. Cx43 was located mainly in plaques on the plasma membrane (white arrowheads). There was more Dlg1 at the plasma membrane (white asterisk) at this time compared to 16 h post-wounding, and co-location with Cx43 could be detected (arrow, merged image). These data suggest that Dlg1 shares a similar pattern of movement with Cx43 during the wound closure process, but changes in Dlg1 location follow those of Cx43. We quantified Cx43 co-localisation with Dlg1 during wound closure. This was done by quantifying co-localisation in all cells in each of five images. Figure 2b shows that there was significant co-localisation at 0 h and there was a statistically significant drop in levels of co-localisation at 16 h post-wounding. Figure 2c shows a significant reduction in Dlg1 co-localisation with Cx43 at 8 and 16 h post-wounding, but some co-localisation remained throughout the time course.

### Dlg1 and Cx43 levels are reduced during wound closure

**Fig. 3:**
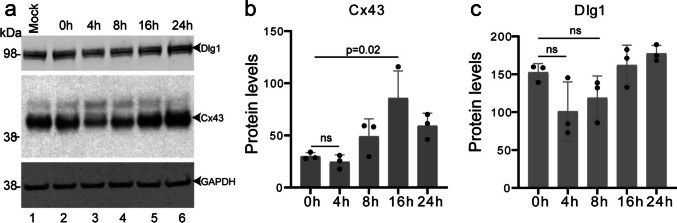
Changes in Cx43 and Dlg1 protein levels during scrape wound closure.** a** Western blots showing levels of Dlg1, Cx43 and GAPDH as a control in scrape-wounded confluent HaCaT cells. **b** Graph of quantification of levels of Cx43 in scrape-wounded confluent HaCaT cells. **c** Graph of quantification of levels of Dlg1 in scrape-wounded confluent HaCaT cells. Graphs show the actual Mean Gray Values of protein bands measured by ImageJ relative to the GAPDH loading controls. The data shown are the mean and the standard deviation from the mean of four separate experiments. ns, not statistically significant

We have previously demonstrated that Dlg1 regulates Cx43 levels in keratinocytes (Scott et al. 2023). Since it has been reported that Cx43 levels decrease during wound closure, we determined how levels of Cx43 and Dlg1 changed over time in the HaCaT scrape wound model. HaCaT keratinocytes were grown to confluence, and three scrape wounds were performed on cell monolayers grown in wells of a 6-well cell culture plate. Cells were harvested, protein lysates were prepared and subjected to western blot analysis. As expected, Cx43 levels were reduced at 4 h post-wounding, although this was not statistically significant perhaps because the majority of cells on the plate were not subjected to scrape wounding. (Fig. 3a, b). By 16 h, Cx43 levels were significantly greater than those observed at the start of the experiment. Dlg1 levels also decreased at 4 and 8 h post-wounding. At 16 h, Dlg1 levels were similar to those in untreated cells (Fig. 3a, c).

### Cx43 and Dlg1 colocalise in cell projections during wound healing in live cell experiments

**Fig. 4:**
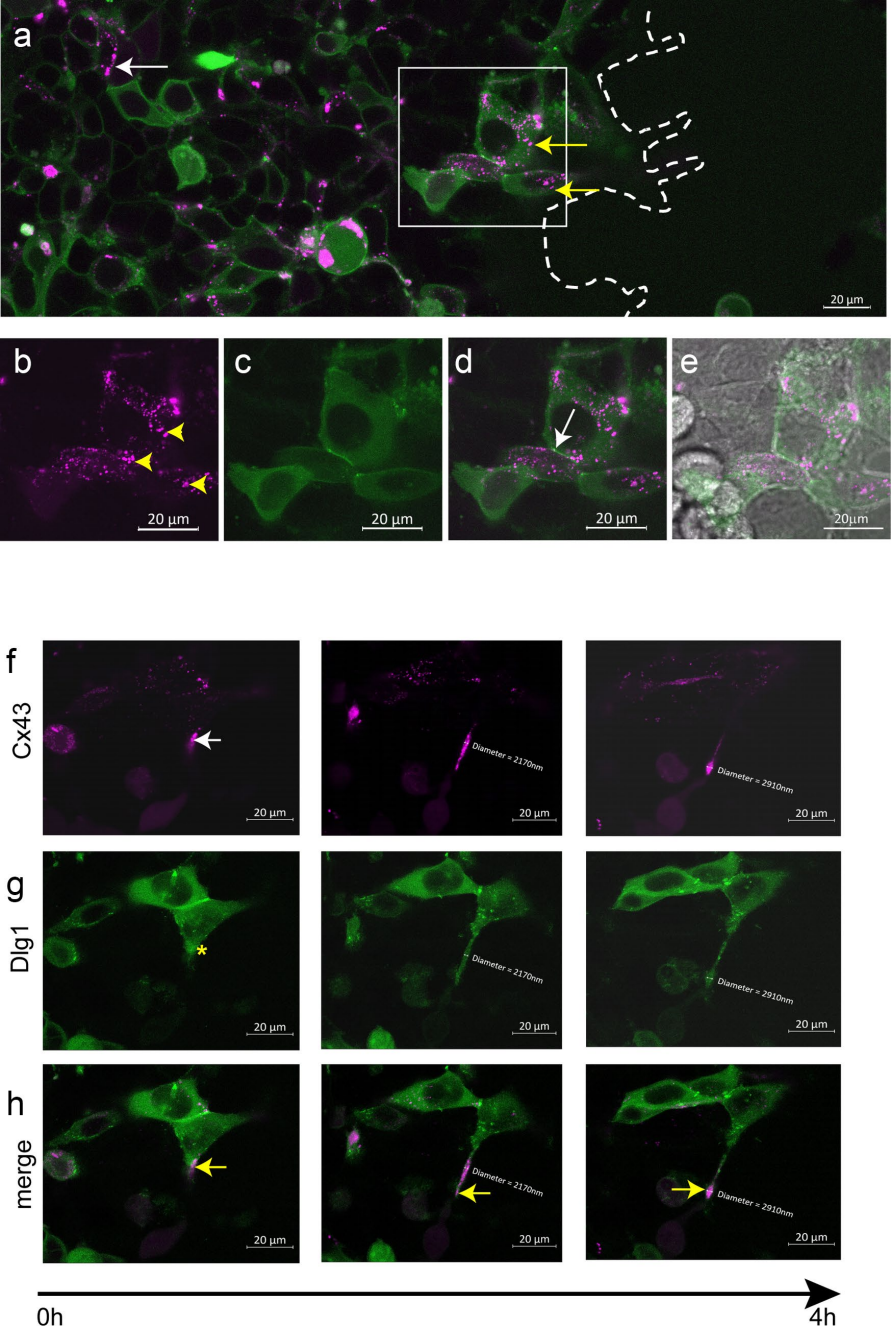
Cx43 and Dlg1 relocate to the cytoplasm and co-locate in cell projections in cells migrating towards the wound edge.** a** An image from live cell confocal immunofluorescence microscopy of HEK293 cells transfected with pmCherry-Cx43 (magenta) and pDlg1-GFP (green) at the edge of a scrape wound. Only a small number of cells co-expressed both proteins. The wound edge is indicated with a dashed line. Scale bar = 20 µm. The white box indicates cells shown in (**b**–**e**). **b** Cx43 antibody staining (magenta) in cells migrating towards the wound edge and co-expressing Cx43 and Dlg1. Arrowheads indicate Cx43 puncta in the cytoplasm. **c** Dlg1 staining (green) in cells migrating towards the wound edge and co-expressing Cx43 and Dlg1. **d** Merged image of Cx43 and Dlg1 staining in (**b** and **c**). The arrow indicates Cx43 localized with Dlg1 on the plasma membrane. **e** Phase contrast image of the area showing the Cx43 and Dlg1 co-expressing cells. Scale bars = 20 µm. **f**–**h** Images from a time-lapse live cell imaging experiment showing growth of a cell projection at the wound edge originating from a cell expressing fluorescently-tagged Cx43 and Dlg1 over a 4-h time period. Images show Cx43-mCherry (magenta) (**f**), Dlg1-GFP (green) (**g**) and merged images in HEK293 (H) cells co-expressing the proteins. The white arrow in (**f**) indicates Cx43 in the forming projection. The yellow asterisk in (**g**) indicates Dlg1 position in the cell projection as it begins to form. Yellow arrows in (**h**) indicate co-location of Cx43 and Dlg1 in the cell projection. The diameter of the projection over time is shown. Scale bars = 20 µm

Next, to further examine a spatial relationship between Dlg1 and Cx43 during wound closure, HEK293 cells were transfected with plasmids for the expression of fluorescently tagged mCherry-Cx43 and GFP-Dlg1. Scratch wound assays were performed followed by live cell imaging of the wound area at intervals of 10 min using a Zeiss Axio Observer Z1 microscope (Fig. 4). HEK293 cells were used instead of HaCaT cells due to their higher transfection efficiency. In cells migrating to the wound-edge (Fig. 4a, dotted line) that expressed both tagged proteins, Cx43 (magenta) and Dlg (green) were mostly localised in the cytoplasm (Fig. 4a, yellow arrows). Cx43 was present in puncta (Fig. 4b, yellow arrowheads) while Dlg1 had a diffuse cytoplasmic location (Fig. 4c). At cell–cell junctions, there was still some Cx43 and Dlg1 co-localisation on the plasma membrane (Fig. 4d, white arrow). In cells away from the wound edge, Cx43 and Dlg1 were present on the plasma membrane in co-expressing cells (Fig. 4a, white arrow). Time-lapse imaging over a 4 h time period (Fig. 4f–h) revealed formation of cell projections known to be involved in the wound healing process (Mattila and Lappalainen 2008; Zurzolo 2021; Radstake et al. 2023). Cx43 seemed to move first into the extending projections (Fig. 4f, white arrow) followed by Dlg1 (Fig. 4g, yellow asterisk). During the growth of the projections, Dlg1 moved and colocalised with Cx43 in co-expressing cells (Fig. 4h, yellow arrows). Measurement of these protrusions revealed many were around 2 µm in diameter, suggesting that these are pseudopodia.

### Knockdown of Dlg1 inhibits wound healing in HaCaT cells

**Fig. 5:**
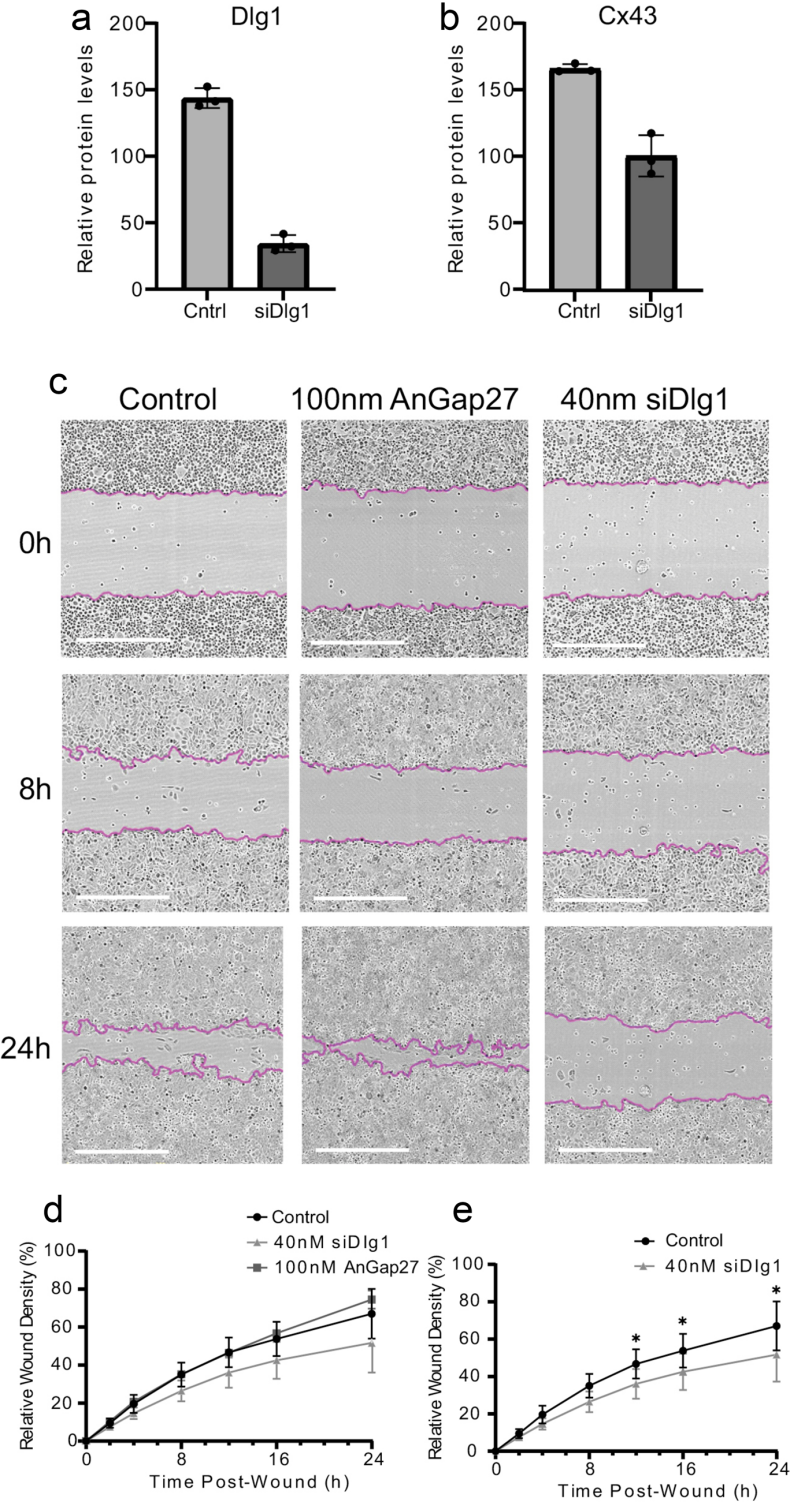
Wound closure is delayed upon Dlg1 depletion. **a** Graph showing levels of Dlg1 in control and siRNA Dlg1-treated HaCaT cells. **b** Graph showing levels of Cx43 in control and Dlg1 siRNA-Dlg1 treated HaCaT cells. **c** Images from an Incucyte wound closure experiment at 0, 8 and 24 h post-scratch wounding. Control, untreated HaCaT cells. siDlg, cells transfected with 40 nM siRNA against Dlg1 24 h prior to wounding. 100 nM AnGap27, cells treated with AnGap27 30 min prior to scratch-wounding. The edges of the wounds are outlined in purple. Scale bars = 600 µm. **d** Graph of relative wound density (RWD) against time post-wounding in the three conditions shown in (**c**). The mean and standard deviation from the mean is shown. Six biological replicates were used for the siRNA experiments and three biological replicates for the AnGap27 experiments. **e** The same data are shown as in (**d**) but without the 100 nM AnGap27 data to highlight the change in relative wound density due to Dlg1 depletion

To analyse the impact of Dlg1 during the wound healing process, HaCaT cell monolayers with and without siRNA depletion of Dlg1 (40 nM siRNA) were scratch wounded using an Incucyte wound-maker tool and immediately imaged using an Incucyte live cell analyser unit, with images being captured every hour (Radstake et al. 2023). This concentration of siRNA reduced Dlg1 levels by 73% compared with control HaCaT cells (Fig. 5a). A 73% reduction in Dlg1 levels resulted in a 40% reduction in Cx43 levels (Fig. 5b). HaCaT cells pre-treated for 30 min with 100 nM AnGap27, a stable analogue of the Cx43 mimetic peptide Gap27, were included as a positive control. This Gap27 analogue has previously been shown to improve scratch wound healing rates (Faniku et al. 2018). The images (Fig. 5c) were collated and analysed to quantify changes in relative wound density (RWD), which is a percentage value representing the relative density of cells within the wound area compared with outside of the wound area at different time points (Fig. 5d, e).

AnGap27-treated cells closed the wound faster than control cells, as expected (Fig. 5c, d). In contrast, cells transfected with siRNA against Dlg1 exhibited significantly reduced RWD compared to control HaCaT cells at the 12–24 h timepoints, suggesting Dlg1 depletion had a deleterious effect on the wound healing process (Fig. 5c, e). The largest differences between the treatment groups were observed at 24 h post-wounding, where 40 nM siDlg1-treated cells had a 15.4% lower RWD compared to control cells (51.7% vs 67.1%), while AnGap27-treated cells had a 7.5% higher RWD compared to control cells (74.6% vs 67.1%), although this was found not to be statistically significant likely due to the low dose of AnGap27 used (Fig. 5d) (Faniku 2018). Figure 5e highlights the data in Fig. 5d comparing control cells with cells treated with 40 nM siDlg1.

### Knockdown of Dlg1 inhibits cell proliferation in HaCaT cells

**Fig. 6:**
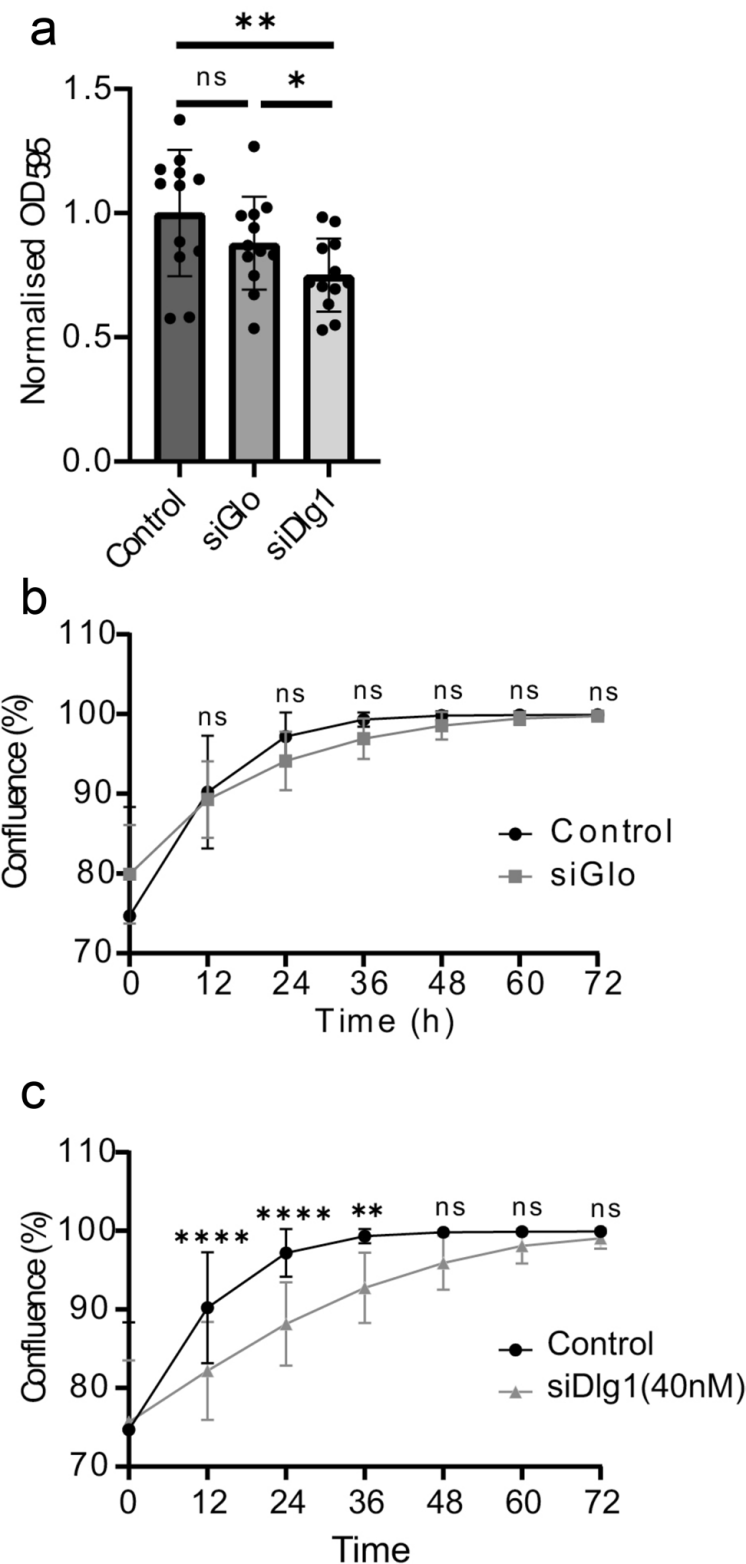
Changes in cell confluence in scrape wounds due to Dlg1 depletion using an MTT assay.** a** HaCaT cells were untreated or were treated 24 h after seeding with a control siRNA (siGLO) or with siRNA targeting Dlg1 and were incubated for a further 24 h. MTT solution was added, and cells were incubated for 3 h at 37 °C. Following dissolution of MTT formazan crystals, OD_595_ values were determined. Values were normalised against control HaCaT cells. Twelve biological replicates were included for each treatment group, with a separate transfection being carried out for each sample. *p < 0.05, **p < 0.01, ns = not significant. **b**–**c** Percentage confluence of control-transfected HaCaT cells and cells transfected with control siRNA (siGLO) (**b**) or siDlg1 (**c**) over a 72 h period. The graph shows the mean and standard deviation from the mean. Twelve samples were included for each treatment group. ns = not significant, **p < 0.01, ***p < 0.001, ****p < 0.0001

Cell proliferation and migration are both important drivers of the wound healing process (Bártolo et al. 2022). With the significant difference in relative wound density observed in control HaCaT cells and siDlg1-treated cells, it was crucial to determine whether this result was due to an effect on cell proliferation, cell migration, or a combination of the two. First, we used an MTT assay on HaCaT cells treated with siDlg1 for 24 h as an indication of whether Dlg1 knockdown affected cell proliferation. OD_595_ values gave a measurement of cellular metabolic activity, which is widely used as an indicator of cell proliferation (Colombo et al. 2017; Kim et al. 2020) (Fig. 6a). All data were normalised to untreated HaCaT control cells to determine any effects on cell metabolism. The results showed that treatment with a control siRNA (siGlo: 40 nM) resulted in a decrease of 12% in cellular metabolic activity, but this was not statistically significant. Treatment with 40 nM siDlg1 resulted in a significant 25% decrease in cellular metabolic activity compared to control cells. This implies that Dlg1 may enhance cell proliferation during wound closure.

To confirm the anti-proliferative effect of treating HaCaT cells with siDlg1, a cell proliferation assay was carried out using the Incucyte live cell analyser unit. Confluence of cells was quantified over time and compared between control, siGlo and siDlg1-treated groups (Fig. 6b–c). Treatment with control siRNA resulted in a 3% decrease in cell confluence at the 24 h timepoint (not statistically significant; Fig. 6b). In contrast, a 10% decrease in cell confluence was observed for cells treated with siRNA against Dlg1, with this difference being statistically significant at the 12, 24 and 36 h timepoints (Fig. 6c).

### Reduced wound healing rate in siDlg1-treated cells is not due to changes in migration

**Fig. 7:**
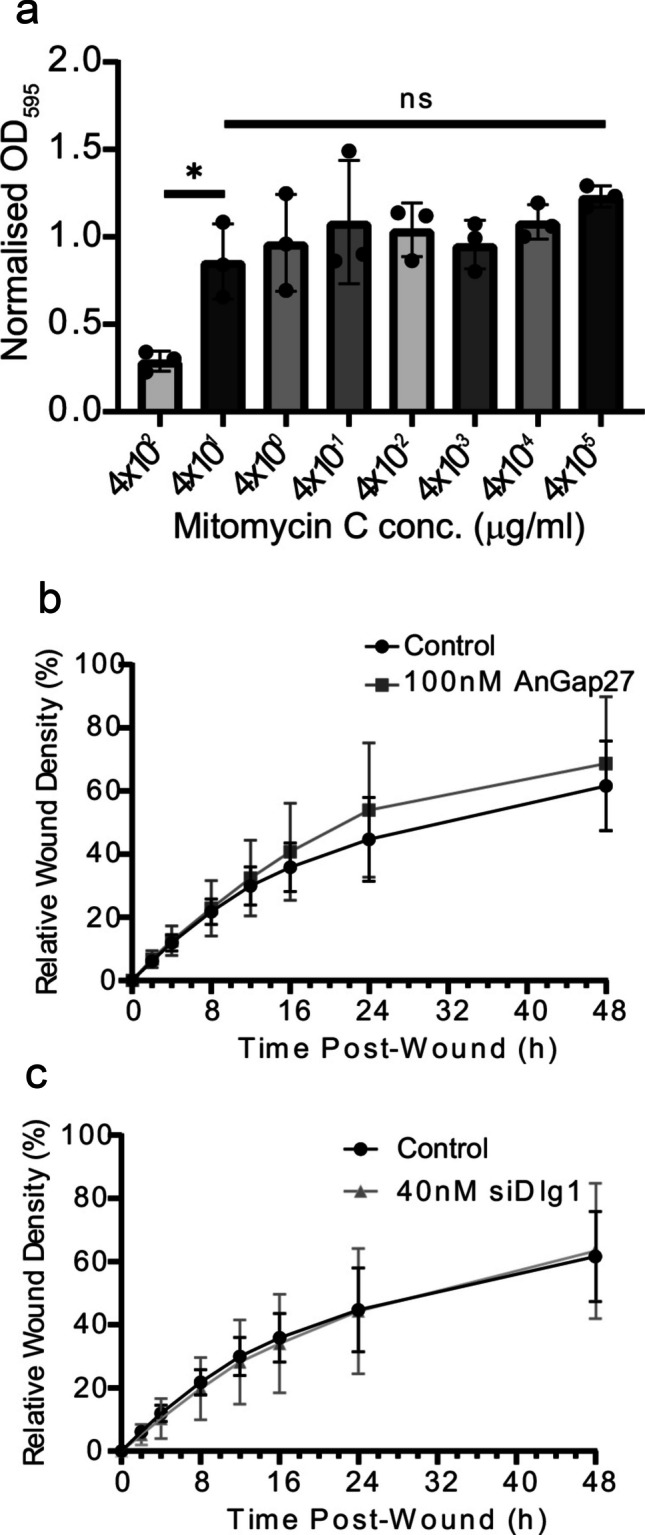
Dlg1 regulates keratinocyte proliferation during wound closure.** a** Assessment of optimum concentration of mitomycin C for use in scratch wound assays was carried out using an MTT assay following mitomycin C treatment. Drug treatment was for 5 min at the concentrations indicated on the *x*-axis. Cells were washed twice with PBS and incubated in fresh media for 24 h at 37 °C. *n* = 3 for each treatment group. **b** Relative wound density (RWD) of untreated HaCaT cells or HaCaT cells transfected with 40 nM siDlg1 and scratch-wounded. Wound density was followed over 48 h. **c** RWD of control HaCaT cells and cells pre-treated with 100 nM AnGap27. Graphs show the mean and standard deviation from the mean. Six biological replicates were used for the siRNA experiments and three biological replicates for the AnGap27 experiment. No statistically significant differences were obtained between values in the graphs in (**b** or **c**)

An experiment to determine the optimum concentration of mitomycin C (MMC) for HaCaT cell scratch assays was carried out using an MTT assay (Fig. 7a). Treatment with log-fold decreasing concentrations of MMC (4 × 10^2^–4 × 10^−5^ µg/ml) was carried out for 5 min. Cells were washed twice in PBS and incubated in fresh medium for 24 h. Following this, an MTT assay was performed. Optical density readings were taken at a wavelength of 595 nm to determine cell viability. Control samples were included in the MTT assay to represent the number of viable cells in test samples before the addition of MMC. The results of test samples were normalised against control values to give a measure of viable cells in comparison to the starting number of cells, with a value of one signifying an identical quantity of viable cells before and after a 24-h incubation following MMC treatment. Reduced cell viability was apparent at a concentration of 4 × 10^2^ µg/ml MMC, with cell viability reduced by 71% compared with control samples. However, cell viability was improved in cells treated with 4 × 10^1^ µg/ml MMC. The highest concentration of MMC which blocked cell proliferation without affecting cell viability was 4 × 10^−1^ µg/ml. Therefore, this concentration of MMC was used in experiments in Fig. 7b and c.

Scratch wound assays were carried out as before with the inclusion of a 5-min pre-treatment with 4 × 10^−1^ mg/ml MMC to block cell proliferation and allow assessment of changes in cell migration alone (Fig. 7b, c). When comparing the MMC-treated groups with the previous scratch wound assay, it took twice as long for the MMC-treated groups to reach similar relative wound densities as the non-MMC-treated groups, representing the contribution of cell proliferation to the wound healing process (compare Figs. 5c to 7 c). At the final timepoint following MMC-treatment, 100 nM AnGap27-treated cells had a higher RWD compared to the control HaCaT cell group (68.7% compared to 61.6%), but this was not statistically significant (Fig. 7b). This suggests the effect of AnGap27 is conserved irrespective of cell division and therefore is due to increased cell migration, which is supported by previous studies on Gap27 (Faniku et al. 2018). In contrast to the previous data, MMC-treated cells transfected with siRNA against Dlg1 did not show a significantly different RWD compared to control cells (Fig. 7c). This indicates that cell migration was unaffected by Dlg1 depletion, suggesting that the differences observed when comparing relative wound density of control and siDlg1-treated cells in the experiment in Fig. 5 were due to a decrease in cell proliferation.

## Discussion

AlphaFold3 modelling of the structures of the C-termini of Cx43 and Dlg1 indicated three main areas of interaction. The regions in sites A and C had many contact sites and low predicted error in the relative positions of the interacting residues. The contacts in site B were predicted with less confidence and fewer interacting residues. Site A (aa 263–269) has previously been shown to interact with Drebrin (Butkevich et al. 2004; Singh and Lampe 2003) and c-Src/PTEN/Csk (Jaraíz-Rodríguez et al. 2017; González-Sánchez et al. 2016). Site B (aa 283–288) has been shown to interact with NEDD4 and AP-2 (Spagnol et al. 2016, Fong et al. 2013). Site C (aa 302–320) has not previously been demonstrated to be involved in protein–protein interactions. In agreement with our previous biochemical data, the terminal four amino acids of Cx43 were not found to interact with Dlg1 (Macdonald et al. 2012). This modelling also supports our previous biochemical data that showed the SH3, HOOK and GUK domains of Dlg1 were involved in interaction with Cx43 (Macdonald et al. 2012). Our modeling provides guidance for future empirical evaluation of the validity of these predicted interaction sites and a possible future test of whether site A can interact simultaneously with Dlg1, Drebrin and c-Src/PTEN/Csk, or if binding is mutually exclusive.

Cx43 relocation from the cell membrane to the cytoplasm was observed during the early phases of wound healing, where loss of membrane Cx43 has been shown to facilitate cell migration (Lorraine et al. 2015). Dlg1 is a binding partner of Cx43, and the proteins interact at the plasma membrane of keratinocytes (Macdonald et al. 2012; Scott et al. 2023). Moreover, Dlg1 controls levels of Cx43 in keratinocytes and is required to maintain Cx43 at the plasma membrane (Scott et al. 2023). We demonstrated in this current study that Dlg1 also relocated to the cytoplasm and some Dlg1 co-located with Cx43 in the cytoplasm of cells at the leading edge of scrape wounds during wound resolution, although this may simply be due to its diffuse cytoplasmic location. At later times, Cx43 reappeared on the plasma membrane followed by Dlg1. The best-known partner protein of Cx43 in regulating wound closure is ZO-1 (Ambrosi et al. 2016, Gourdie et al. 2006, Hunter et al. 2005; Rhett et al. 2010; Zheng et al. 2019). However, the Dlg1-interacting region of Cx43 is not located in the terminal four amino acids of the C-terminal domain that bind to ZO-1 (Macdonald et al. 2012). Therefore, the relationship between Cx43 and Dlg1 during wound closure must be different from that between Cx43 and ZO-1.

Dlg1 maintains adherens junctions in keratinocytes through the Scribble complex and SGEF, a Rho guanidine exchange factor (Awadia et al. 2019; Bonello et al. 2019; Laprise et al. 2004). Dlg1 is also known to interact indirectly with the actin cytoskeleton (Firestein and Rongo 2001; Reuver and Garner 1998). Adherens junctions are removed from the plasma membrane during wound closure, and the actin cytoskeleton is extensively remodelled (Ebstrup et al. 2021), which could lead to Dlg1 internalisation. Since adherens junctions and F-actin aid in delivery of Cx43 to the plasma membrane (Meyer et al. 1992), and Dlg1 seems to maintain Cx43 at the plasma membrane (Scott et al. 2023), wound closure-related cellular remodelling could be the cause of internalisation of Cx43. We did not investigate Cx43 localisation upon Dlg1 depletion during wound resolution because both processes cause loss of Cx43 from the plasma membrane, leading to a cytoplasmic location. In addition, both wounding and Dlg1 depletion cause decreased Cx43 levels, which could compromise determining localisation of any residual Cx43.

The importance of Dlg1 in wound closure was shown in siRNA depletion experiments where loss of Dlg1 led to reduced wound closure over time compared to control siRNA treatment. Dlg1 was subsequently shown to regulate the proliferation of keratinocytes in scrape wounds. A function for Dlg1 in enhancing proliferation is contrary to its proposed role as a tumour suppressor (James and Roberts 2016). However, there is evidence that Dlg1 can have both positive and negative effects on cell growth. For example, Dlg1 interacts with SGEF (RhoG-specific guanine nucleotide exchange factor) to enhance RhoG activity and thereby promote cell proliferation and migration (Krishna Subbaiah et al. 2012). Dlg1 is also recruited to the midbody between daughter cells during cell division, and loss of Dlg1 in these circumstances can result in arrest during cytokinesis (Unno et al. 2008). On the other hand, Dlg1 is well known to regulate cell polarity, which was not assessed in this study. Therefore, Dlg1 may enhance cellular proliferation by facilitating both cell division and changes in cell polarity that stimulate wound resolution.

Both Cx43 and Dlg1 were localised to cellular protrusions in live cell experiments, which may represent pseudopodia due to their diameter of 2–3 µm. Cx43 is present in pseudopodia, likely due to its association with actin (Ochalski et al. 1995; Ambrosi et al. 2016). Dlg1 plays a role in actin polymerisation (Round et al. 2005) and is associated with cellular protrusions (Iizuka-Kogo et al. 2005; Sharma et al. 2006) including lamellipodia (Xavier et al. 2004). Thus, Dlg1 may regulate Cx43 signaling in cellular protrusions in addition to conventional gap junction signaling, with these playing an important role during the process of wound closure.

## Conclusions

A major limitation of this study is the lack of confirmation of the AlphaFold3-predicted Cx43/Dlg1 molecular interactions by mutational analyses. However, our scratch wound assay results using HaCaT keratinocytes provide evidence that Dlg1 functions as a positive component of the natural wound closure process. This could be through a role for Dlg1 in enhancing cell proliferation. However, as a binding partner of Cx43, Dlg1 could regulate Cx43 presence at the plasma membrane as we have previously demonstrated in normal keratinocytes. Dlg1 was found to colocalize with Cx43 in cell projections during wound closure. Although we have shown previously that Dlg1 is required to maintain Cx43 on the plasma membrane, the role of Dlg1 in cell shape determination could indicate an indirect regulation of Cx43 recycling from the plasma membrane during wound closure via changes in the actin cytoskeleton.

## Supplementary Information

Below is the link to the electronic supplementary material.

Supplementary Material 1: Validation of the Cx43/Dlg1 AlphaFold3 Model. **a**. Confidence in the relative placement of amino acids was assessed by predicted aligned error (PAE) values generated by AlphaFold3. Areas which are the same colour are predicted to be in the correct positions relative to each other, while areas which are different colours are not predicted to be in the correct relative positions. Sites A and C of Cx43 are predicted to be in the correct positions relative to each other and the corresponding interacting areas of Dlg1. Site **b** is not predicted to be in the correct position relative to the corresponding interacting area of Dlg1. **b**–**e**. ProSA validation of the Cx43 and Dlg1 models. The local model quality of Cx43 (**b**) and Dlg1 (**c**) was assessed by plotting energies of individual amino acid residues. Energies are shown as an average value of both 40 residue (dark green line) and 10 residue (light green line) fragments, as plotting individual residue energies results in large fluctuations which are difficult to interpret. Positive energy values correspond to residues which are more likely to be incorrect. The overall model quality of Cx43 (**d**) and Dlg1 (**e**) was determined by comparing the model z-score (black dot, value shown on bottom left-hand corner of the graph) to the z-score of experimentally determined protein structures from the Protein Data Bank (PDB, light blue dots = X-ray structures, dark blue dots = NMR structures). Accurate models should have comparable z-scores to experimentally determined structures of roughly equivalent amino acid length. **f**–**g**. ProCHECK validation of the Cx43/Dlg1 model. A Ramachandran plot (**f**) shows the phi and psi bond angles of each amino acid, grouped into regions which are energetically more (red/brown areas) or less (dark yellow/light yellow) favourable. High-quality protein models have large proportions of amino acid residues in the energetically favoured and allowed regions. Conversely, residues in disallowed regions indicate steric hinderance and are less likely to be found in native protein structures, suggesting placement of these residues may be inaccurate. **g.** Summary table of the Ramachandran Plot. (PNG 776 KB)
High Resolution Image (TIF 16.1 MB)

## Data Availability

The underlying data for the experiments in this manuscript can be accessed at: [10.5525/gla.researchdata.1947] (https://www.eur03.safelinks.protection.outlook.com/?url=https%3A%2F%2Fdoi.org%2F10.5525%2Fgla.researchdata.1947&data=05%7C02%7CSheila.Graham%40glasgow.ac.uk%7Cf08b178218dd4600d5a308dd7db528c6%7C6e725c29763a4f5081f22e254f0133c8%7C1%7C0%7C638804939232617372%7CUnknown%7CTWFpbGZsb3d8eyJFbXB0eU1hcGkiOnRydWUsIlYiOiIwLjAuMDAwMCIsIlAiOiJXaW4zMiIsIkFOIjoiTWFpbCIsIldUIjoyfQ%3D%3D%7C0%7C%7C%7C&sdata=X0bZXDQGK6wGfr4vII1t%2Ft%2Ft3mYl09gVFB3JXllfk80%3D&reserved=0).
