# Supplementary figures and images for: The relationship between Connexin 43 (Cx43) and partner protein, human discs large homologue-1 (Dlg1) during wound closure in keratinocytes
Source: Cell Tissue Res. 2026 Feb 18;403(2):23. doi: 10.1007/s00441-025-04030-9 (PMC12913269; doi:10.1007/s00441-025-04030-9)

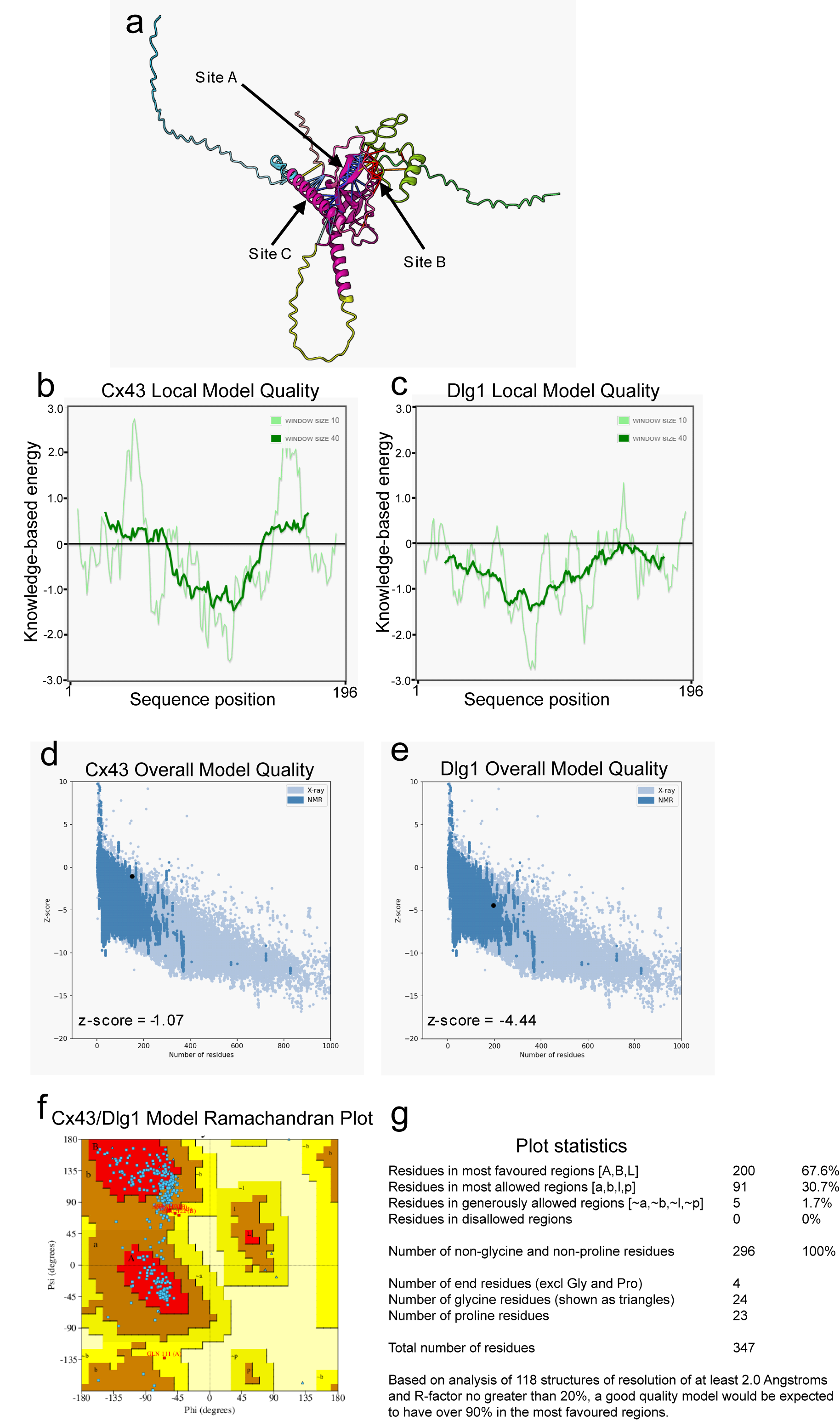

Supplement: Supplementary file 1 — Supplementary Material 1: Validation of the Cx43/Dlg1 AlphaFold3 Model. a. Confidence in the relative placement of amino acids was assessed by predicted aligned error (PAE) values generated by AlphaFold3. Areas which are the same colour are predicted to be in the correct positions relative to each other, while areas which are different colours are not predicted to be in the correct relative positions. Sites A and C of Cx43 are predicted to be in the correct positions relative to each other and the corresponding interacting areas of Dlg1. Site b is not predicted to be in the correct position relative to the corresponding interacting area of Dlg1. b–e. ProSA validation of the Cx43 and Dlg1 models. The local model quality of Cx43 (b) and Dlg1 (c) was assessed by plotting energies of individual amino acid residues. Energies are shown as an average value of both 40 residue (dark green line) and 10 residue (light green line) fragments, as plotting individual residue energies results in large fluctuations which are difficult to interpret. Positive energy values correspond to residues which are more likely to be incorrect. The overall model quality of Cx43 (d) and Dlg1 (e) was determined by comparing the model z-score (black dot, value shown on bottom left-hand corner of the graph) to the z-score of experimentally determined protein structures from the Protein Data Bank (PDB, light blue dots = X-ray structures, dark blue dots = NMR structures). Accurate models should have comparable z-scores to experimentally determined structures of roughly equivalent amino acid length. f–g. ProCHECK validation of the Cx43/Dlg1 model. A Ramachandran plot (f) shows the phi and psi bond angles of each amino acid, grouped into regions which are energetically more (red/brown areas) or less (dark yellow/light yellow) favourable. High-quality protein models have large proportions of amino acid residues in the energetically favoured and allowed regions. Conversely, residues in disallowed regions [file 441_2025_4030_MOESM1_ESM.png]
